# Case Report: Residual Atrial Shunt Lesions in Aging Adults With Congenital Heart Disease: An Underestimated Risk of Stroke?

**DOI:** 10.3389/fcvm.2022.847244

**Published:** 2022-03-16

**Authors:** Matthias Schneider, Varius Dannenberg, Bernd Opgen-Rhein, Felix Berger, Burkert Pieske, Harald Gabriel, Leif-Hendrik Boldt

**Affiliations:** ^1^Department of Internal Medicine and Cardiology, Charité Universitätsmedizin Berlin, Berlin, Germany; ^2^German Heart Center Berlin, Berlin, Germany; ^3^Department of Internal Medicine II, Cardiology, Medical University of Vienna, Vienna, Austria; ^4^Department of Pediatric Cardiology, Charité Universitätsmedizin Berlin, Berlin, Germany; ^5^German Centre for Cardiovascular Research (DZHK), Berlin, Germany; ^6^Berlin Institute of Health, Berlin, Germany

**Keywords:** embolic stroke, adults with congenital heart disease, echocardiography, cardioembolic and cryptogenic stroke, Ebstein anomaly, ASD, case report

## Abstract

We report two cases of paradoxical cerebral embolism in adults with congenital heart disease (ACHD) with residual atrial shunt lesions, a 59 year-old male patient with partial detachment of a surgical ASD closure patch, and a 57 year-old male patient with Ebstein’s anomaly and a large patent foramen ovale. Considering these mechanisms and the increasing incidence of venous thrombosis with age, a higher prevalence of paradoxical embolism in ACHD patients with residual atrial shunts may be suspected. Regular follow-up of patients with ACHD remains important throughout life even in seemingly stable lesions.

## Introduction

The number of aging adults with congenital heart disease (ACHD) is constantly growing, as are late sequelae in this cohort (1). The risk for ischemic stroke is eleven times higher in young patients with CHD when compared to controls (2), the cumulative risk between the ages of 18 and 64 years is 6–8% (3). The majority of events occur in patients with cyanotic defects and in those without sinus rhythm (4). Arrhythmias are common in ACHD patients (5), in particular there is a high prevalence of atrial arrhythmias (6, 7). While paradoxical embolism *via* a patent foramen ovale (PFO) is associated with cryptogenic stroke in the general population and PFO closure reduces the rate of subsequent events in selected patients (8–10), up to now cryptogenic stroke has not been investigated systematically in ACHD patients.

## Case Presentation

**FIGURE 1:**
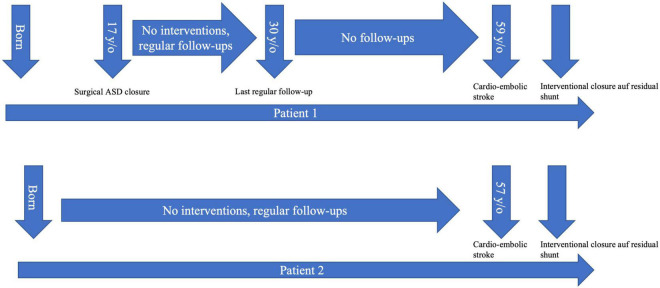
Timeline depicting the medical history of both presented patients. ASD, atrial septum defect.

**FIGURE 2:**
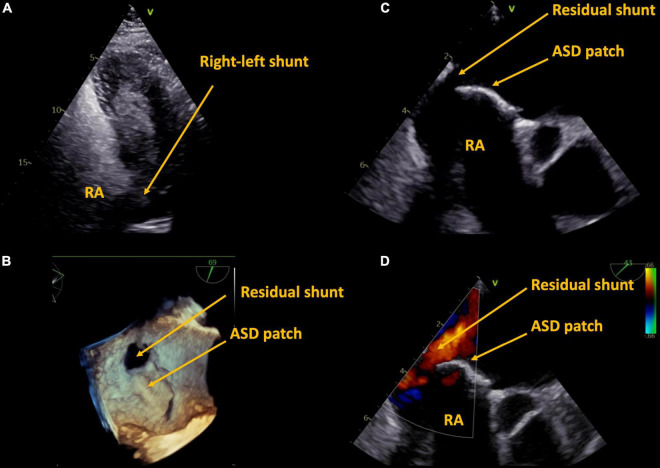
Case 1, 59 year-old patient with a history of surgical ASD closure with partial detachment of the patch. Residual shunt shown by right-heart contrast agent **(A)**, and by transesophageal echocardiography in 3D **(B)**, 2D **(C)**, and 2D color **(D)**. ASD, atrial septum defect, RA, right atrium.

We report two cases of paradoxical cerebral embolism in ACHD patients with residual atrial shunt lesions (Figure 1). The first patient is a 59 year-old male who had received surgical repair of secundum atrial septum defect (ASD) 42 years ago. There were no regular follow-ups after the age of 30, his further medical history was unremarkable, exercise capacity was excellent. The patient presented to our hospital with acute embolic stroke. Transesophageal echocardiography revealed a residual atrial septal defect in the posterior inferior region due to partial detachment of the surgical patch (Figure 2) with spontaneous transfer of ultrasound contrast agent from right to left. The right ventricle was of borderline size, calculated Qp:Qs was 1.4:1. In absence of other reasons for embolic stroke (non-smoker, no dyslipidemia, no arterial hypertension, no history of atrial fibrillation, and unremarkable carotid ultrasound), paradoxical embolism was assumed and interventional closure of the residual atrial septum defect was performed with a 25 mm septal occluder device.

**FIGURE 3:**
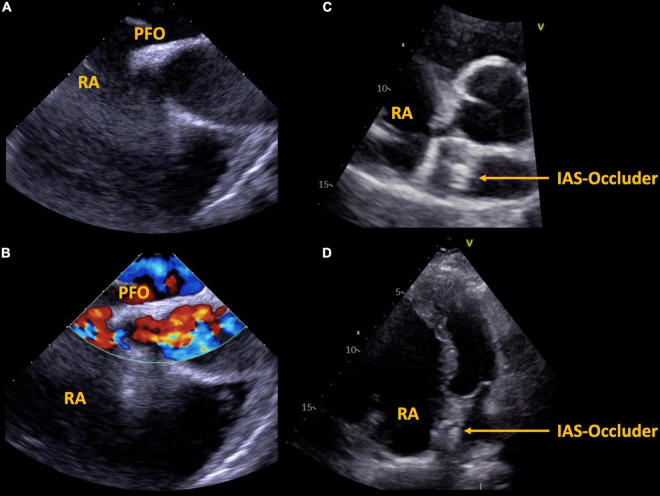
Case 2, 57 year-old patient with Ebstein’s anomaly. A large PFO is shown via transesophageal echocardiography without **(A)** and with **(B)** color Doppler imaging. Interventional septal occluder implantation was performed, the result is shown in panels **(C,D)**. PFO, patent foramen ovale, IAS, inter atrial septum, RA, right atrium.

The second patient is a 57 year-old male with known Ebstein’s anomaly. Exercise capacity had been unchanged for years as were right heart dimensions. This patient also presented with embolic stroke. The patient was a non-smoker, there was no history of dyslipidemia, arterial hypertension, or atrial fibrillation, carotid ultrasound was unremarkable). TEE revealed a large PFO with spontaneous transfer of ultrasound contrast agent from right to left (Figure 3). Paradoxical embolism was assumed and the PFO was closed interventionally with a 25 mm PFO closure device. Exercise capacity remained unchanged after PFO closure.

The two presented cases of paradoxical embolism illustrate that mechanisms of stroke in ACHD patients can be different from those of the general population (e.g., high right atrial pressure in Ebstein’s anomaly, large shunt in partial detachment of ASD patch). Considering these mechanisms and the increasing incidence of venous thrombosis with age (11), a higher prevalence of paradoxical embolism in ACHD patients with residual atrial shunts may be suspected and should be investigated in prospective trials.

## Conclusion

With the increasing number of aging ACHD patients, late sequelae not directly related to the initial congenital heart disease significantly influence their morbidity and mortality. Regular follow-up of patients with ACHD remains important throughout life even in seemingly stable lesions.

## Data Availability Statement

The original contributions presented in the study are included in the article/supplementary material, further inquiries can be directed to the corresponding author.

## Author Contributions

MS: conceptualization and visualization. MS and VD: writing – original draft preparation. HG, VD, FB, BO-R, BP, and L-HB: writing – review and editing. BP, HG, and L-HB: supervision. All authors have read and agreed to the published version of the manuscript.

## Conflict of Interest

HG was consultant for ABBOTT Medical and GORE Medical. The remaining authors declare that the research was conducted in the absence of any commercial or financial relationships that could be construed as a potential conflict of interest.

## Publisher’s Note

All claims expressed in this article are solely those of the authors and do not necessarily represent those of their affiliated organizations, or those of the publisher, the editors and the reviewers. Any product that may be evaluated in this article, or claim that may be made by its manufacturer, is not guaranteed or endorsed by the publisher.
